# Associations between seminal plasma osteopontin level and sperm motility in infertile men with asthenozoospermia

**DOI:** 10.3389/fendo.2025.1487650

**Published:** 2025-05-19

**Authors:** Peng Zhang, Yifu Leng, Yuanxin Liu, Jiayi Liu, Chaoran Li, Jie Qin

**Affiliations:** ^1^ Department of Urology, The First Affiliated Hospital of Dalian Medical University, Dalian, China; ^2^ Department of Reproduction Medicine Center, The Affiliated Zhongshan Hospital, Dalian University, Dalian, China; ^3^ Medical Department, The First Affiliated Hospital of Dalian Medical University, Dalian, China

**Keywords:** male infertility, asthenozoospermia, osteopontin, seminal plasma, sperm motility

## Abstract

**Purpose:**

Male infertility, a complex multifactorial condition, is frequently caused by asthenozoospermia (AZS). Although osteopontin (OPN) has been implicated in mammalian reproduction, its specific effects on sperm motility and fertility are not well understood. This study investigates the relationships between seminal plasma OPN levels and sperm parameters in cases of male infertility.

**Methods:**

A total of 158 semen samples were analyzed, comprising 78 from infertile men with AZS and 80 from healthy fertile controls. OPN concentrations in seminal plasma were measured using the ELISA method. Additionally, we assessed the *in vitro* effect of OPN on sperm motility parameters in AZS patients and controls.

**Results:**

Significantly lower OPN concentrations were observed in the seminal plasma of infertile men with AZS compared to healthy controls (*P*<0.0001). OPN levels discriminated between the groups, with an area under the curve of 0.793. Additionally, *in vitro* OPN treatment significantly improved sperm motility in the AZS group, enhancing progressive and total motility, as well as kinematic parameters in a concentration-dependent manner.

**Conclusions:**

This study established a link between OPN level and sperm motility in infertile individuals with AZS, suggesting the potential of OPN as a biomarker for AZS and as a supplement for assisted reproductive techniques.

## Introduction

Infertility is a significant global public health issue, impacting approximately 15% of couples in their reproductive years (1). It is estimated that male factors contribute to 30-50% of these cases (2). The etiology of male infertility is multifaceted, often linked to a reduction in sperm count or anomalies in sperm morphology, which can hinder the sperm’s ability to reach and fertilize an egg (3). These issues may stem from a variety of sources, including testicular dysfunction, endocrine disorders, lifestyle influences, congenital anatomical anomalies, exposure to gonadotoxins, and the effects of aging (3, 4). Among the leading causes of male infertility is asthenozoospermia (AZS), characterized by reduced sperm motility (5). Despite ongoing research, effective treatments for idiopathic AZS remain elusive, and the underlying mechanisms contributing to AZS and broader male infertility issues are largely unidentified.

During ejaculation and deposition into the female genital tract, spermatozoa are transported by seminal plasma, a mixture of secretions primarily from male urogenital tract glands (6). Seminal plasma is generally recognized to support sperm survival and fertility across various species, containing proteins, mRNA and metabolites (7). The proteins play pivotal roles in regulating semen coagulation and liquefaction and promoting sperm motility and fertilization (8, 9). The functionality of seminal plasma is believed to be linked to the properties of individual proteins, which can occur either as components bound to the sperm membrane or as free entities within the plasma (10, 11). The identification of specific proteins and their contribution to either the positive or negative effects observed in seminal plasma could provide insights into potential markers and therapeutic targets for AZS and male infertility (6, 12).

Osteopontin (OPN, also known as secreted phosphoprotein 1) is a versatile extracellular matrix phosphoprotein found across various tissues and fluids (13, 14), including the reproductive systems (15, 16). It serves as a crucial molecule for cell adhesion and migration, capable of binding to multiple ligands such as αvβ3 integrin, certain CD44 isoforms, and fibronectin (14, 17). OPN involves various physiological and pathological processes, including bone resorption, metastasis, inflammation, cellular immunity, tissue repair, and mammalian reproduction (18–21). Animal studies have demonstrated that OPN expression in the testis, epididymis, and ductus deferens is intimately linked with spermatogenesis and sperm function (22–24). OPN has been identified at 55 and 25 kDa molecular weights in cauda epididymal fluid and testicular parenchyma homogenates, respectively (25). Furthermore, *in vitro* fertilization studies have revealed that sperm treated with an OPN antibody exhibited reduced fertilization rates and an increased incidence of polyspermy compared to those treated with a control medium (20), suggesting OPN’s role in facilitating fertilization and preventing polyspermy. Despite these insights, the role of OPN in human male reproduction, particularly its expression and impact on sperm quality and fertility, remains inadequately explored.

In this study, we initially evaluated the concentrations of OPN in the seminal plasma of infertile men with AZS and healthy fertile men. Comprehensive analyses of the associations between OPN levels and sperm parameters were performed. Additionally, *in vitro* experiments were conducted to assess the impact of OPN on sperm motility in both infertile men with AZS and healthy fertile controls. Through a methodical evaluation of seminal OPN concentrations and their association with sperm motility, this study intends to offer new insights into the pathophysiology of AZS and explore its potential as both a diagnostic biomarker and a supplement for assisted reproductive techniques.

## Materials and methods

### Participants

A total of 158 participants (78 infertile men with AZS and 80 healthy fertile men) were enrolled from undergoing routine sperm analysis for couple infertility or pre-pregnancy counselling at the reproductive center of the Affiliated Zhongshan Hospital of Dalian University between June 2020 and July 2022. The demographics and sperm parameters of all the participants are summarized in **Table 1**. The infertile men were described as unable to father children via natural conception for more than one year of unprotected intercourse but still had a chance to fertilize via assisted reproductive technology. Meanwhile, AZS is diagnosed as <32% of progressively motile sperm with more than 4% normal sperm morphology by the analysis in the andrology laboratory more than two times at different times. Infertile men with varicocele, cryptorchidism, obstruction of the vas deferens, endocrine hypogonadism, immune infertility (positive anti-sperm antibodies), samples with leukocytospermia (>1 ×10^6^ white blood cells/mL), acute or chronic inflammation and cancer were excluded from this study. Andrology laboratory tests showed that the α-glucosidase levels (mU per ejaculate) in the semen and testosterone levels in the serum were within the normal range (α-glucosidase level ≥ 20 mU per ejaculate; testosterone levels ≥ 12.1 nmol/L) of all the infertile men with AZS. The healthy fertile men as the control group are proven fertility. Participants who used drugs that affected sperm parameters were excluded, as were those who suffered from malignant tumor, mental or psychological problems, severe smoking and alcohol habits, substance abuse, or hazardous occupations. This study has received approval from the Ethics Committee of the Affiliated Zhongshan Hospital of Dalian University (approval number: 2019326) in compliance with the Declaration of Helsinki and pertinent guidelines. Written informed consent was obtained from all participants.

**Table 1:** Demographics and sperm parameters of the participants.

Characteristics	HFM	AZS	*P-*value ^c^
Number	80	78	NS
Age (years) ^a^	33.44 ± 5.7	34.5 ± 4.7	NS
Sexual abstinence time (days) ^a^	4.2 ± 1.8	4.0 ± 1.6	NS
α-Glucosidase (mU per ejaculate)	NA	45.35 ± 10.1	*-*
Testosterone (nmol/L)	NA	16.32 ± 3.9	*-*
Sperm parameters			
Progressive motility (%) ^a^	37.06 ± 3.5	18.47 ± 8.1	*P*<0.0001
Total motility (%) ^a^	40.14 ± 4.4	20.29 ± 8.6	*P*<0.0001
Concentration (×10^6^/mL) ^a^	79.7 ± 39.8	68.03 ± 41.6	NS
Total sperm count (×10^6^) ^b^	249.7 ± 24.5	220.9 ± 20.6	NS
SPR (%) ^a^	11.9 ± 7.4	4.95 ± 4.4	*P*<0.0001
VCL (mm/s) ^a^	23.67 ± 5.5	11.28 ± 5.3	*P*<0.0001
VSL (mm/s) ^a^	11.08 ± 2.5	5.15 ± 2.5	*P*<0.0001
VAP (mm/s) ^a^	15.8 ± 2.9	7.67 ± 3.6	*P*<0.0001
Normal morphology (%) ^a^	4.88 ± 2.3	4.12 ± 3.3	NS
WBCs in semen	<1×10^6/^mL	<1×10^6/^mL	NS

^a^Data are expressed as mean ± SD. ^b^Data are expressed as mean ± SEM. ^c^P values are generated by the Mann–Whitney U-test.

HFM, healthy fertile men; AZS, asthenozoospermia; NS, not significant; NA, not analyzed. SPR, super-progressive ratio; VCL, curvilinear velocity; VSL, straight line velocity; VAP, average path velocity; WBCs, white blood cells.

### Semen collection and sperm motility analysis

The semen samples from the participants were obtained by masturbation after sexual abstinence for 2–7 days. After liquefaction, one portion of the sperm sample was put on a computer-assisted sperm analysis system (CASA, CASAS-SSA-II, Suijia, Beijing, China) and evaluated the sperm concentration, volume and motility by routine procedures. A minimum of five different fields, each containing more than 200 spermatozoa, were assessed to obtain sperm concentration, motility, and kinematic parameters such as curvilinear velocity (VCL), straight-line velocity (VSL), average path velocity (VAP) and super-progressive ratio (SPR; VAP>30.0 μm/s, VSL/VAP ≥ 0.8, and VSL/VCL≥ 0.5). Semen samples meeting the following criteria were classified as normal: volume ≥ 1.5 mL, pH ≥ 7.2. Sperm morphology was evaluated according to the 5th WHO criteria using a microscope (ZEISS, Germany). Two independent observers counted the percentage of normal morphology sperm for each sample with three replicates. Seminal plasma from the remaining semen was separated by centrifugation at 3000rpm for 15 min. Then the seminal plasm samples were stored at -80°C for further analysis.

### Seminal plasma OPN measurement

According to the manufacturer’s instructions, seminal plasma OPN concentration was evaluated using the Human Osteopontin (OPN) Quantikine ELISA Kit (R&D systems, Minneapolis, MN, USA, catalog number DOST00). The concentration of OPN in the seminal plasma was finally determined by comparing the OD values of the samples to the standard curves. The analytical sensitivity of the assay was 0.024 ng/mL. The intra-assay coefficients of variation for the kits were 8.4% (AZS) and 7.9% (healthy fertile controls), and the inter-assay variation coefficients were 9.2% (AZS) and 8.7% (healthy fertile controls).

### OPN treatment and sperm motion parameters

An *in vitro* study was designed to evaluate the effects of OPN on sperm motility. Semen samples were collected from 38 men undergoing infertility counselling, which included 20 men diagnosed with AZS and 18 healthy fertile controls. Post-liquefaction at 37°C for one hour, the sperm were washed third with Ham’s F-10 supplemented with HEPES, using centrifugation at 500g for five minutes to remove seminal fluid. The sperm pellets were then homogenized by gentle vertexing and inspected microscopically to ensure the absence of germ cells and leukocytes. All sperm samples were assessed and confirmed to exhibit normal morphology.

Sperm samples from both AZS patients and healthy controls were co-incubated for 1–2 hours with varying concentrations of recombinant human OPN (R&D Systems, Minneapolis, MN, USA; catalog no. 1433-OP)—including a blank control, 0.01 μg/mL, 0.1 μg/mL, and 0.5 μg/mL—in Ham’s F-10 medium supplemented with HEPES and 5% bovine serum albumin (BSA). Recombinant OPN was reconstituted in sterile phosphate-buffered saline (PBS) prior to use. Sperm motility parameters were subsequently assessed using CASA. Comparative analyses were performed across different OPN concentrations and incubation durations, focusing on the proportions of progressively motile and total motile sperm, as well as kinematic parameters including VCL, VSL, VAP and SPR.

### Statistical analysis

Quantitative data were expressed as mean ± standard deviation (SD) or standard error of the mean (SEM) for each group. We analyzed group differences using the non-parametric Mann-Whitney U test. Spearman’s correlation analyses were used to evaluate the relationships between the demographic, sperm parameters, and the expression levels of five clock genes. Receiver operating characteristic (ROC) curves and areas under the curves (AUC) were used to evaluate the ability of clock genes to distinguish between infertile men with AZS and healthy fertile men. Statistical analyses were performed with the SPSS software version 17.0 (SPSS Inc., Chicago, IL, USA). A *P*-value < 0.05 was considered statistical significance.

## Results

### Characteristics of demography and sperm parameters of the participants

This study involved the collection of 158 semen samples, comprising 78 from infertile men with AZS and 80 from healthy fertile men for the evaluation of OPN. Demographic details and sperm parameters-encompassing progressive and total motility, total count, concentration, SPR, VCL, VSL, VAP and normal morphology were summarized in **Table 1**. No significant differences were observed in age, duration of sexual abstinence, sperm concentration, total sperm count, normal morphology and white blood cell number in semen between the two groups. However, both progressive and total motility percentages were substantially lower in the AZS group (18.47 ± 8.1 and 20.29 ± 8.6, respectively) compared to the healthy fertile group (37.06 ± 3.5 and 40.14 ± 4.4, respectively, *P*<0.0001). Similarly, sperm SPR, VCL, VSL, and VAP values were significantly reduced in the AZS group compared to the healthy group (*P*<0.0001).

### The concentration of OPN in the seminal plasma of infertile men with AZS patients

**Figure 1:**
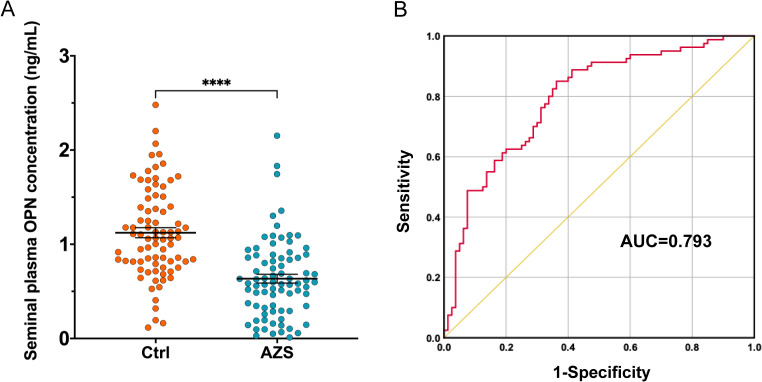
OPN concentration in the seminal plasma of infertile men with AZS (n=78) compared to the fertile men (n=80). **(A)** Scatter plots of OPN concentration in the seminal plasma of infertile men with AZS patients and fertile group using ELISA method. Data are presented as mean ± SEM; ∗∗∗∗*P* < 0.0001. **(B)** ROC curves assessing the discriminative capability of OPN levels between infertile men with AZS and healthy fertile men, showing significant discriminative power with an AUC value of 0.793 (95% Confidence Interval, 0.724–0.863).

The concentration of OPN in the seminal plasma was assessed through ELISA in both infertile men diagnosed with AZS and healthy fertile men. The assay’s analytical sensitivity was established at 0.024 ng/mL, with all participant samples registering above this threshold. The findings revealed a notable decrease in seminal plasma OPN concentration among the AZS patients (Mean ± SEM, 0.635 ± 0.42 ng/mL) when compared to the healthy fertile group (Mean ± SEM, 1.123 ± 0.48 ng/mL, *P*<0.0001, **Figure 1A**).

### The performance of OPN level for the discriminability between the infertile men with AZS and healthy fertile men

The discriminative capability of seminal plasma OPN levels between infertile men diagnosed with AZS and healthy fertile controls was assessed using the AUC derived from ROC curve analysis. The analysis indicated that the OPN levels possess significant discriminative power, with an AUC value of 0.793 (95% Confidence Interval, 0.724–0.863, *P*<0.0001, **Figure 1B**).

### Associations between the OPN level and the sperm parameters

**Figure 2:**
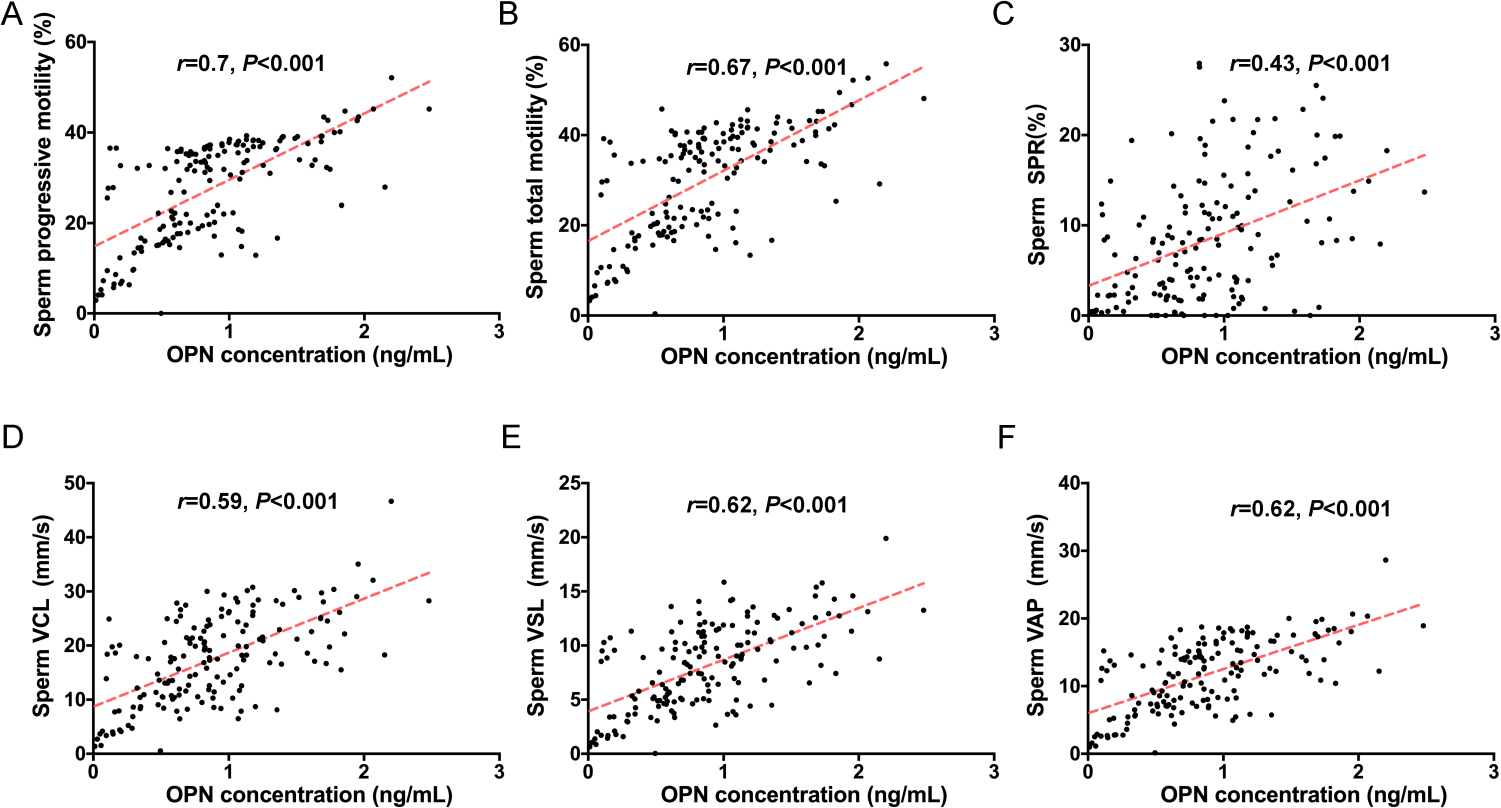
Correlations between seminal plasma OPN levels and sperm motility. Significant correlations were observed between OPN concentration and **(A)** progressive motility (*r*=0.7, *P*<0.01), **(B)** total motility (*r*=0.67, *P*<0.01), **(C)** SPR (*r*=0.43, *P*<0.01), **(D)** VCL (r=0.59, *P*<0.001), **(E)** VSL (r=0.62, *P*<0.001) and **(F)** VAP (r=0.62, *P*<0.001) based on Spearman’s correlation analysis. Red lines indicate linear regression fits.

**Figure 3:**
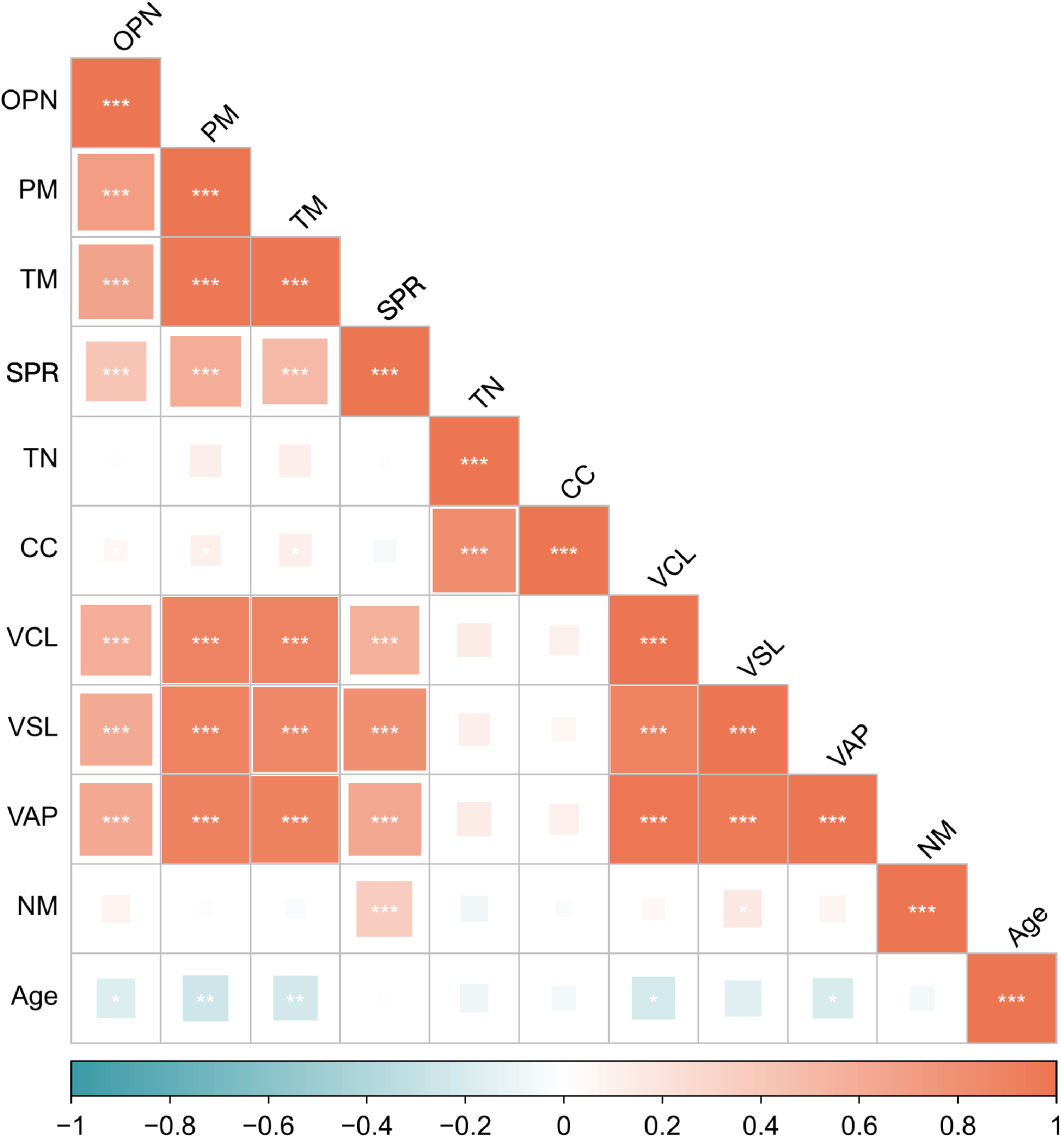
Spearman’s correlations between the seminal OPN level and the sperm parameters. The values of the color bar reflect the correlation coefficients between the seminal OPN level and the sperm parameters. PM, progressive motility; TM, total motility; SPR, super-progressive ratio; TN, total number; CC, concentration; VCL, curvilinear velocity; VSL, straight line velocity; VAP, average path velocity; NM, normal morphology. *P< 0.05, **P< 0.01, ***P< 0.001.

To investigate the potential role of seminal plasma OPN in sperm function, we conducted correlation analyses between OPN concentrations and various sperm parameters across the entire study cohort. Notably, OPN levels exhibited strong positive correlations with both progressive motility (*r* = 0.700, *P* < 0.01) and total motility (*r* = 0.666, *P* < 0.01) (**Figures 2A, B**, **3**). In addition, moderate positive correlations were observed between OPN and parameters related to sperm velocity and movement quality, including SPR (*r* = 0.429, *P* < 0.01, **Figures 2C, E**), VCL (*r* = 0.595, *P* < 0.01, **Figure 2D**), VSL (*r* = 0.617, *P* < 0.01), and VAP (*r* = 0.622, *P* < 0.01, **Figures 2F**, **3**). These findings suggest that higher OPN concentrations may contribute to enhanced sperm motility and kinematic performance.

In contrast, no significant associations were found between OPN levels and sperm concentration or total sperm count, indicating that OPN’s effects may be more specific to motility rather than sperm quantity (**Figure 3**).

We also assessed correlations with potential confounding factors. OPN levels were not significantly associated with participant age or duration of sexual abstinence. However, age showed an inverse correlation with several motility-related parameters, including progressive motility (*r* = –0.248, *P* < 0.01), total motility (*r* = –0.229, *P* < 0.01), VCL (*r* = –0.215, *P* < 0.01), and VAP (*r* = –0.191, *P* < 0.05) (**Figure 3**), supporting prior evidence of age-related declines in sperm motility. No significant relationships were observed between age and sperm SPR, concentration, total count, or normal morphology.

### OPN concentration-dependently activated human sperm motility *in vitro*

**Figure 4:**
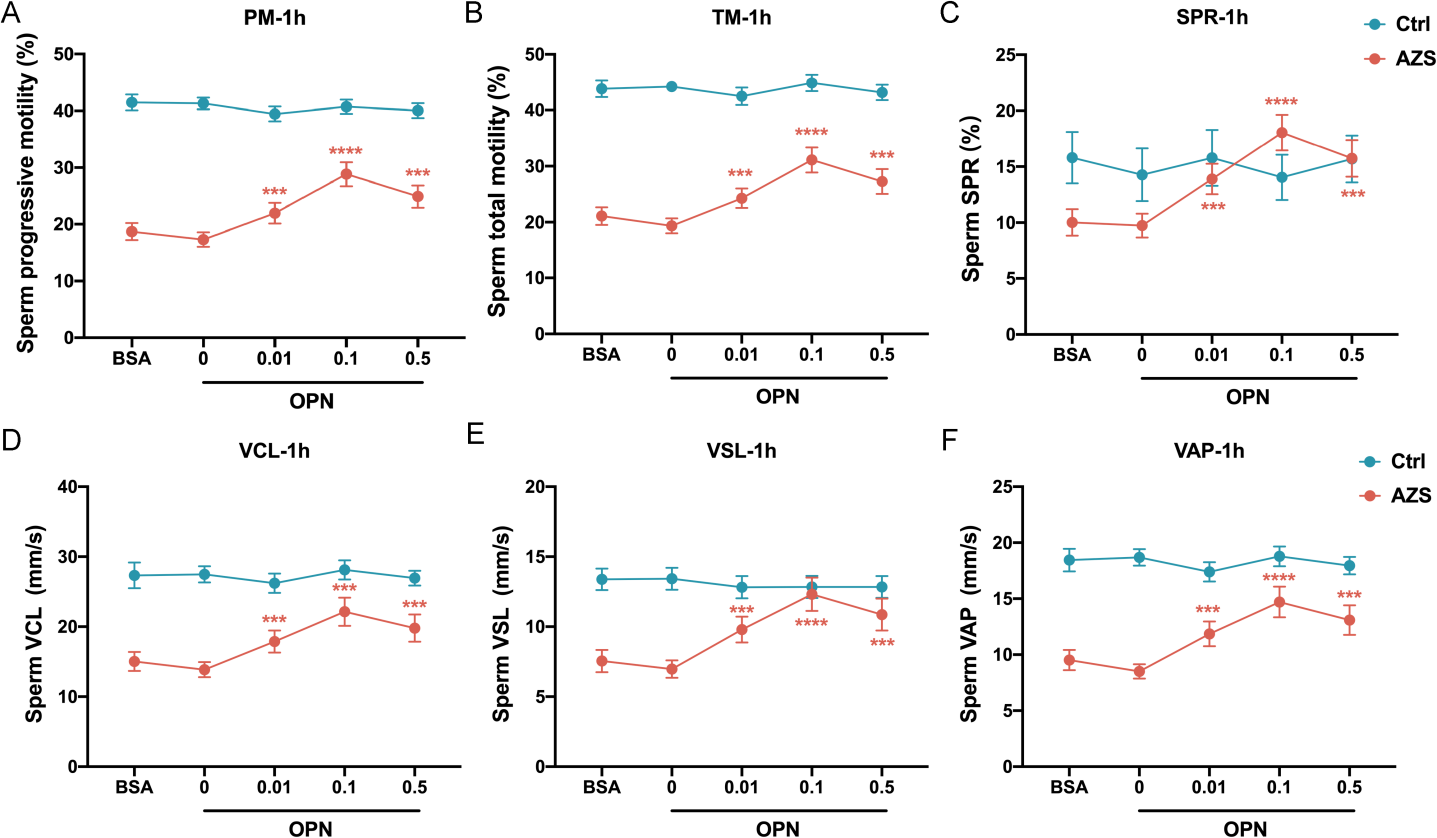
Concentration-dependent activation of human sperm motility by OPN over 1 hour *in vitro*. Following 1 hour of exposure to varying concentrations of OPN, there was a significant enhancement in **(A)** progressive motility, **(B)** total motility, **(C)** SPR, **(D)** VCL, **(E)** VSL, and **(F)** VAP in the AZS group. Data are presented as mean ± SD; Asterisks indicate statistical significance relative to the blank control (BSA group): ∗∗∗*P* < 0.001, ∗∗∗∗*P* < 0.0001,.

**Figure 5:**
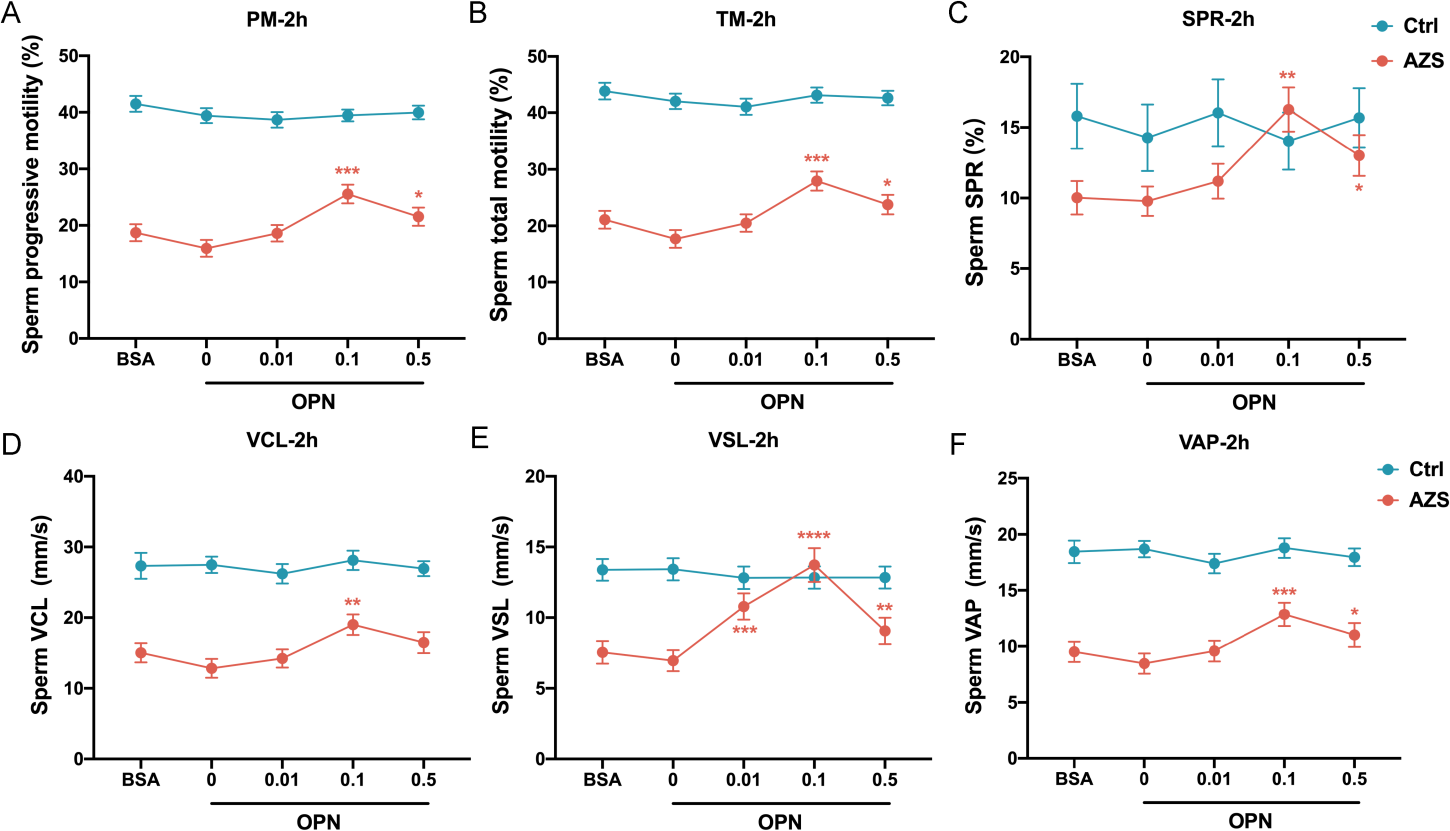
Concentration-dependent activation of human sperm motility by OPN over 2-hour *in vitro*. Notable increases were observed in **(A)** progressive motility, **(B)** total motility, **(C)** SPR, **(D)** VCL, **(E)** VSL, and **(F)** VAP within the AZS group, with effects being concentration-dependent and most pronounced at 0.1μg/mL OPN. Data are presented as mean ± SD; Asterisks indicate statistical significance relative to the blank control (BSA group): ∗*P* < 0.05, ∗∗*P* < 0.01, ∗∗∗*P* < 0.001, ∗∗∗∗*P*<0.0001.

To further explore the association between OPN concentration and sperm motility, sperm samples from men diagnosed with AZS (n=20) and healthy fertile controls (Ctrl, n=18) were incubated with different concentrations of OPN protein (0.01μg/mL, 0.1μg/mL, 0.5μg/mL) for either 1 or 2 hours. Subsequently, sperm motion parameters in them were assessed. After 1h of *in vitro* culture, a significant increase in various sperm motility functions (including progressive motility, total motility, SPR, VCL, VSL and VAP) was observed in the AZS group, with effects being concentration-dependent and most pronounced at 0.1μg/mL OPN (**Figures 4A–F**). This trend persisted with 2 h of exposure (**Figures 5A–F**). Specifically, exposure to 0.1μg/mL OPN increased the progressive motility by 23.12% after 1 hour (**Figure 4A**) and 24.12% after 2 hours (**Figure 5A**), respectively, while the enhancement of total motility was 25.12% after 1 hour (**Figure 4B**) and 26.12% after 2 hours (**Figure 5B**), correspondingly. Moreover, OPN treatment notably increased the sperm SPR in the AZS group, achieving the highest values of 2.16 μg/mL after 1 hour (**Figure 4C**) and 2.36 μg/mL after 2 hours (**Figure 5C**) at a concentration of 0.1μg/mL. In contrast, sperm motility in healthy fertile men remained unchanged across all OPN concentrations and incubation periods (**Figures 4A–F**, **5A–F**), indicating that the response to OPN is particularly pronounced in sperm with impaired motility.

To further investigate how OPN affects sperm motion, we assessed whether OPN treatment altered the kinematic parameters of sperm motility. The analysis revealed significant enhancements in VCL, VSL, and VAP with OPN treatment for both 1 hour (*P*<0.001, **Figures 4D–F**) and 2 hours (*P*<0.001, **Figures 5D–F**), compared to the control group. These findings indicate that OPN substantially improves both the velocity and linearity of movement in sperm exhibiting poor motility.

**Figure 6:**
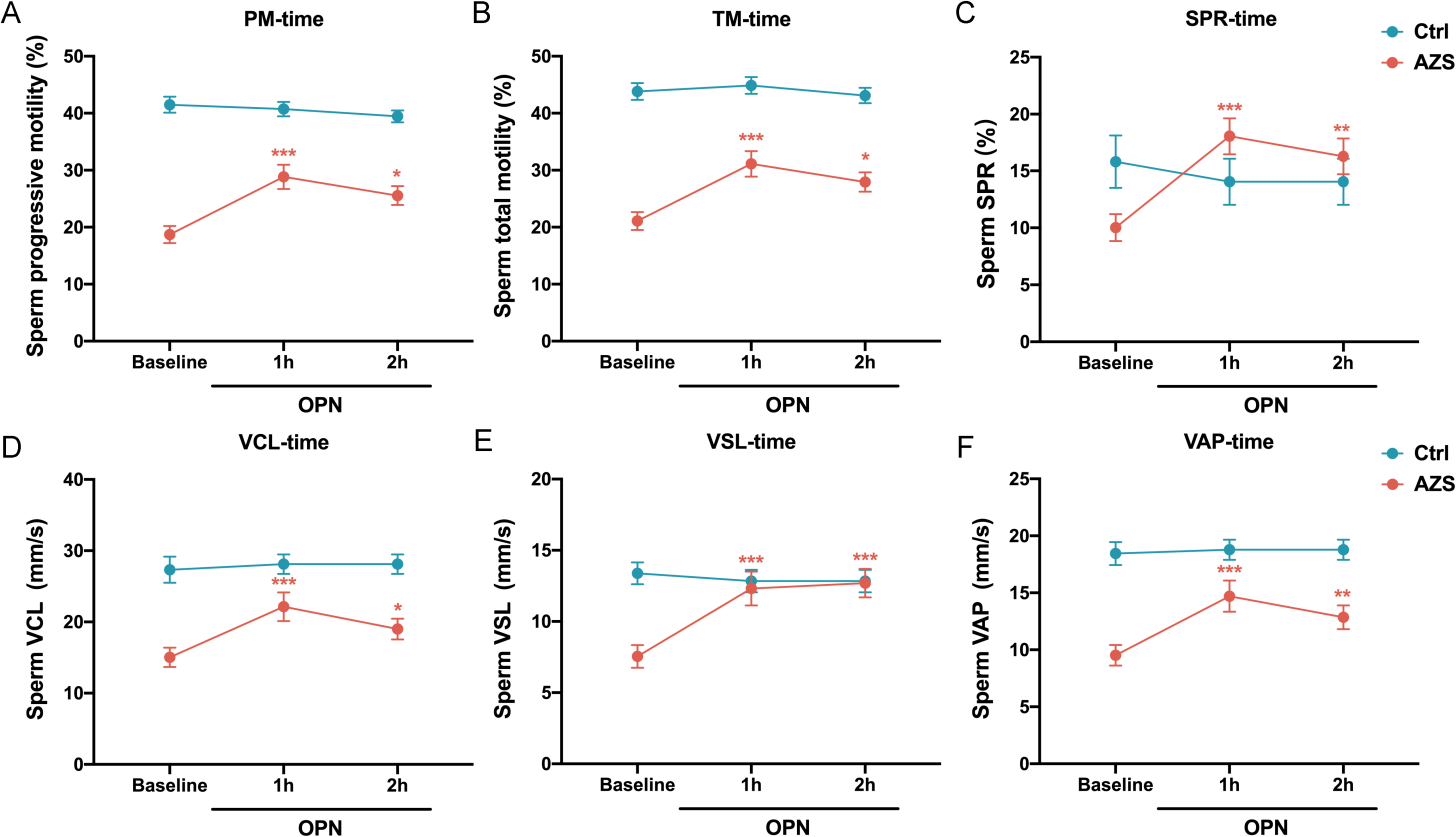
Effects of OPN on sperm motility parameters over different incubation time. Enhancements were significantly more pronounced after 1 hour of incubation than after 2 hours across various parameters: **(A)** progressive motility, **(B)** total motility, **(C)** SPR, **(D)** VCL, **(E)** VSL, and **(F)** VAP. Data are presented as mean ± SD; Asterisks indicate statistical significance relative to the baseline group: ∗*P* < 0.05, ∗∗*P* < 0.01, ∗∗∗*P* < 0.001.

Additionally, we investigated the effect of OPN on sperm motility parameters over different incubation periods. Analysis revealed that sperm motility functions, including progressive motility, total motility, SPR, VCL, VSL, and VAP, were more significantly enhanced after 1 hour of incubation than 2 hours (**Figures 6A–F**). This suggests that a 1-hour incubation with a concentration of 0.1μg/mL OPN yields optimal improvements in sperm motility parameters, highlighting the potential therapeutic value of OPN in enhancing sperm motility in AZS.

## Discussion

Our findings demonstrate a marked decrease in OPN concentrations in the seminal plasma of men afflicted with AZS compared to those in healthy fertile controls, aligning with prior animal research highlighting OPN’s critical role in male reproductive functions. The significant correlations between OPN levels and sperm motility parameters substantiate the hypothesis that OPN could play a role in sperm functionality and fertility. The discernible difference in OPN levels between the groups, along with its association with improved sperm motility metrics observed in *in vitro* treatments, suggests that OPN not only serves as a potential biomarker of fertility status but may also provide benefits for assisted reproduction.

Seminal plasma is increasingly recognized as a valuable clinical sample for the non-invasive diagnosis of male reproductive system disorders (6, 26). Recent studies highlighted a significant variability in the protein composition of the seminal plasma between fertile and infertile men (27). The role of seminal plasma is critical during ejaculation, where it not only transports spermatozoa but also facilitates the binding of seminal proteins to the sperm surface, potentially influencing sperm transport and survival within the female genital tract through membrane remodeling (11). A significant number of proteins present in seminal plasma are directly involved in sperm production and maturation and in interactions with the zona pellucida and oocyte fusion (28). For instance, assays based on seminal proteins such as TEX101, ECM1, and ACRV1 are currently available or in the final stages of development for clinical application (29). In a preclinical evaluation using the ELISA method on seminal plasma samples from 805 individuals, TEX101 levels were found to be significantly lower in males with infertility compared to the fertile controls (12). Furthermore, the protein ECM1, expressed in the epididymis, has been verified and validated as a biomarker for diagnosing various causes of infertility (7). A study employing high-performance liquid chromatography to analyze water- and fat-soluble compounds associated with antioxidant and metabolic processes in seminal plasma has identified distinct biomarkers for differentiating infertile patients from fertile controls (30). Additionally, emerging research on cell-free microRNAs in seminal plasma has revealed a correlation between changes in sperm quality and abnormal levels of miRNA, either in the seminal plasma or in its exosomes.

Several animal studies have highlighted the potential role of OPN in male reproductive functions^20-25^. Initial findings identified OPN in rat testis and epididymal fluids, suggesting its origin as a Sertoli cell product (24). Subsequent research has demonstrated cell- and region-specific expression and regulation of OPN within the epididymis, particularly in the microvilli, small endocytic vesicles, and endosomes, where it may facilitate the removal of calcium from the epididymal lumen, thus preventing mineral accumulation and enhancing sperm fertility (31). Immunofluorescence detection has confirmed the presence of OPN in cauda epididymal spermatozoa (22, 25). Notably, OPN was first identified as a 55 kDa high fertility marker in Holstein bull seminal plasma, produced by the ampulla and vesicular gland (32). A recent study investigating the relationship between sperm quality and OPN levels in Malakli shepherd dogs found significant variability in OPN concentrations across ejaculate fractions, correlating lower levels with reduced sperm motility and morphology (33). In humans, our study observed a marked decrease in OPN levels in the seminal plasma of men with AZS compared to healthy controls. The discriminative power of seminal plasma OPN levels, as demonstrated by ROC curve analysis, validates its utility as a diagnostic marker for differentiating between infertile men with AZS and healthy fertile men. These results suggest that OPN holds promise as a diagnostic and prognostic tool for addressing male reproductive disorders.

The strong correlations between seminal plasma OPN concentrations and key sperm motility parameters—such as progressive motility, SPR, VCL, VSL, and VAP—together with normal morphology, further highlight OPN’s critical role in enhancing sperm motility. Our findings, combined with previous animal studies, suggest that OPN’s varied expression and functionality across species may provide new insights into the mechanisms underlying sperm vitality and fertility. Seminal plasma OPN may enhance sperm motility through multiple complementary mechanisms. As a multifunctional glycoprotein, OPN can act as a cell adhesion and migration molecule by binding to integrins and CD44 receptors on the sperm membrane, thereby promoting membrane stability and facilitating interactions essential for motility (18). In addition, OPN has been shown to modulate calcium signaling pathways and intracellular kinase cascades, which are critical for sperm SPR and flagellar movement. Its role in protecting sperm from oxidative stress and maintaining mitochondrial function may also contribute to improved sperm viability and energy metabolism (31). Collectively, these mechanisms support the hypothesis that elevated levels of OPN in seminal plasma are positively associated with enhanced sperm functionality.

We further performed *in vitro* experiments to reveal a concentration-dependent effect of OPN on sperm motility enhancement, particularly pronounced in the AZS group. This concentration-dependent response to OPN, especially at 0.1μg/mL, underscores the nuanced and potent influence of OPN on sperm motility. The lack of significant changes in sperm motility parameters in healthy fertile men further suggests that OPN’s motility-enhancing effects are most beneficial in cases of compromised sperm motility, such as in AZS. *In vitro* studies on animals also revealed a role for sperm-associated OPN in fertilization and the prevention of polyspermy. For example, bull sperm treated with an OPN antibody resulted in fewer oocytes being fertilized than those treated with a control medium, increasing the incidence of polyspermy (25).

Furthermore, we observed a significant enhancement in multiple sperm motility parameters—including progressive motility, total motility, SPR, VCL, VSL, and VAP—in the AZS group following OPN treatment. These improvements exhibited a concentration-dependent pattern within the range of 0 to 0.1 µg/mL, suggesting an optimal window for OPN-mediated functional enhancement. Notably, a higher concentration of 0.5 µg/mL was associated with a slight reduction in these beneficial effects, which may be attributed to receptor saturation or the activation of negative feedback regulatory mechanisms. Additionally, the partial decline in motility parameters observed at 2 hours post-treatment may reflect increased metabolic demands induced by OPN-stimulated motility, potentially resulting in mitochondrial ATP depletion—particularly in AZS sperm, which may already possess compromised mitochondrial function. However, there remains a possibility that BSA may interact with OPN, potentially modulating the extent or efficiency of OPN’s effects on sperm motility. Further mechanistic studies will be required to clarify whether such interactions occur and how they might influence sperm function. Together, our findings underscore the importance of both timing and concentration in modulating OPN’s effects and suggest its potential utility as an adjunctive agent in assisted reproductive technologies.

In summary, this study underscores the significant role of OPN in sperm motility in the context of AZS-associated infertility. The findings reveal that lower OPN concentrations in the seminal plasma are associated with reduced sperm motility, emphasizing OPN’s potential as a diagnostic biomarker. Furthermore, the enhancement of motility parameters following *in vitro* OPN treatment, supports the potential of OPN as an adjunctive agent for maintaining sperm motility *in vitro*. Future research should focus on elucidating the molecular mechanisms through which OPN exerts its effects on sperm motility and exploring the clinical applicability of OPN-based treatments for improving reproductive outcomes in infertile men.

## Data Availability

The raw data supporting the conclusions of this article will be made available by the authors, without undue reservation.

## References

[1] Sang Q Ray PF Wang L. Understanding the genetics of human infertility. Science. (2023) 380:158–63. doi: 10.1126/science.adf7760 37053320

[2] Agarwal A Mulgund A Hamada A Chyatte MR. A unique view on male infertility around the globe. Reprod Biol Endocrinol. (2015) 13:37. doi: 10.1186/s12958-015-0032-1 25928197 PMC4424520

[3] Krausz C Riera-Escamilla A. Genetics of male infertility. Nat Rev Urol. (2018) 15:369–84. doi: 10.1038/s41585-018-0003-3 29622783

[4] Salas-Huetos A Bullo M Salas-Salvado J. Dietary patterns, foods and nutrients in male fertility parameters and fecundability: a systematic review of observational studies. Hum Reprod Update. (2017) 23:371–89. doi: 10.1093/humupd/dmx006 28333357

[5] Zhang P Li C Gao Y Leng Y. Altered circadian clock gene expression in the sperm of infertile men with asthenozoospermia. J Assist Reprod Genet. (2022) 39:165–72. doi: 10.1007/s10815-021-02375-y PMC8866580 35000095

[6] Drabovich AP Saraon P Jarvi K Diamandis EP. Seminal plasma as a diagnostic fluid for male reproductive system disorders. Nat Rev Urol. (2014) 11:278–88. doi: 10.1038/nrurol.2014.74 24709963

[7] Druart X Rickard JP Tsikis G de Graaf SP. Seminal plasma proteins as markers of sperm fertility. Theriogenology. (2019) 137:30–5. doi: 10.1016/j.theriogenology.2019.05.034 31285051

[8] Onel T Ayla S Keskin I Parlayan C Yigitbasi T Kolbasi B. Leptin in sperm analysis can be a new indicator. Acta Histochem. (2019) 121:43–9. doi: 10.1016/j.acthis.2018.10.006 30482507

[9] Sha Y Liu W Li S Osadchuk LV Chen Y Nie H. Deficiency in AK9 causes asthenozoospermia and male infertility by destabilising sperm nucleotide homeostasis. EBioMedicine. (2023) 96:104798. doi: 10.1016/j.ebiom.2023.104798 37713809 PMC10507140

[10] Dousset B Hussenet F Daudin M Bujan L Foliguet B Nabet P. Seminal cytokine concentrations (IL-1beta, IL-2, IL-6, sR IL-2, sR IL-6), semen parameters and blood hormonal status in male infertility. Hum Reprod. (1997) 12:1476–9. doi: 10.1093/humrep/12.7.1476 9262280

[11] Castillo J de la Iglesia A Leiva M Jodar M Oliva R. Proteomics of human spermatozoa. Hum Reprod. (2023) 38:2312–20. doi: 10.1093/humrep/dead170 37632247

[12] Korbakis D Schiza C Brinc D Soosaipillai A Karakosta TD Legare C. Preclinical evaluation of a TEX101 protein ELISA test for the differential diagnosis of male infertility. BMC Med. (2017) 15:60. doi: 10.1186/s12916-017-0817-5 28330469 PMC5363040

[13] Pardo A Gibson K Cisneros J Richards TJ Yang Y Becerril C. Up-regulation and profibrotic role of osteopontin in human idiopathic pulmonary fibrosis. PloS Med. (2005) 2:e251. doi: 10.1371/journal.pmed.0020251 16128620 PMC1198037

[14] Icer MA Gezmen-Karadag M. The multiple functions and mechanisms of osteopontin. Clin Biochem. (2018) 59:17–24. doi: 10.1016/j.clinbiochem.2018.07.003 30003880

[15] Saklamaz A Calan M Yilmaz O Kume T Temur M Yildiz N. Polycystic ovary syndrome is associated with increased osteopontin levels. Eur J Endocrinol. (2016) 174:415–23. doi: 10.1530/EJE-15-1074 26701868

[16] Cancel AM Chapman DA Killian GJ. Osteopontin localization in the Holstein bull reproductive tract. Biol Reprod. (1999) 60:454–60. doi: 10.1095/biolreprod60.2.454 9916014

[17] Wei J Marisetty A Schrand B Gabrusiewicz K Hashimoto Y Ott M. Osteopontin mediates glioblastoma-associated macrophage infiltration and is a potential therapeutic target. J Clin Invest. (2019) 129:137–49. doi: 10.1172/JCI121266 PMC6307970 30307407

[18] Chai YL Chong JR Raquib AR Xu X Hilal S Venketasubramanian N. Plasma osteopontin as a biomarker of Alzheimer’s disease and vascular cognitive impairment. Sci Rep. (2021) 11:4010. doi: 10.1038/s41598-021-83601-6 33597603 PMC7889621

[19] Hosaka K Rojas K Fazal HZ Schneider MB Shores J Federico V. Monocyte chemotactic protein-1-interleukin-6-osteopontin pathway of intra-aneurysmal tissue healing. Stroke. (2017) 48:1052–60. doi: 10.1161/STROKEAHA.116.015590 PMC5390817 28292871

[20] Hao Y Mathialagan N Walters E Mao J Lai L Becker D. Osteopontin reduces polyspermy during *in vitro* fertilization of porcine oocytes. Biol Reprod. (2006) 75:726–33. doi: 10.1095/biolreprod.106.052589 16870945

[21] Monaco E Gasparrini B Boccia L De Rosa A Attanasio L Zicarelli L. Effect of osteopontin (OPN) on *in vitro* embryo development in cattle. Theriogenology. (2009) 71:450–7. doi: 10.1016/j.theriogenology.2008.08.012 18835636

[22] Zhang GM Lan S Jia RX Yan GY Wang LZ Nie HT. Age-associated and tissue-specific expression of osteopontin in male Hu sheep reproductive tract. Tissue Cell. (2016) 48:496–502. doi: 10.1016/j.tice.2016.07.003 27514848

[23] Souza FF Chirinea VH Martins MI Lopes MD. Osteopontin in seminal plasma and sperm membrane of dogs. Reprod Domest Anim. (2009) 44 Suppl;2:283–6. doi: 10.1111/j.1439-0531.2009.01447.x 19754587

[24] Siiteri JE Ensrud KM Moore A Hamilton DW. Identification of osteopontin (OPN) mRNA and protein in the rat testis and epididymis, and on sperm. Mol Reprod Dev. (1995) 40:16–28. doi: 10.1002/mrd.1080400104 7702867

[25] Erikson DW Way AL Chapman DA Killian GJ. Detection of osteopontin on Holstein bull spermatozoa, in cauda epididymal fluid and testis homogenates, and its potential role in bovine fertilization. Reproduction. (2007) 133:909–17. doi: 10.1530/REP-06-0228 17616721

[26] Guo Y Jiang W Yu W Niu X Liu F Zhou T. Proteomics analysis of asthenozoospermia and identification of glucose-6-phosphate isomerase as an important enzyme for sperm motility. J Proteomics. (2019) 208:103478. doi: 10.1016/j.jprot.2019.103478 31394311

[27] Jeseta M Pospisilova A Mekinova L Franzova K Ventruba P Lousova E. Non-invasive diagnostics of male spermatogenesis from seminal plasma: seminal proteins. Diagnostics (Basel). (2023) 13:2468. doi: 10.3390/diagnostics13152468 37568830 PMC10417070

[28] Cannarella R Barbagallo F Crafa A La Vignera S Condorelli RA Calogero AE. Seminal plasma transcriptome and proteome: towards a molecular approach in the diagnosis of idiopathic male infertility. Int J Mol Sci. (2020) 21:7308. doi: 10.3390/ijms21197308 33022946 PMC7582752

[29] Bieniek JM Drabovich AP Lo KC. Seminal biomarkers for the evaluation of male infertility. Asian J Androl. (2016) 18:426–33. doi: 10.4103/1008-682X.175781 PMC4854096 26975492

[30] Lazzarino G Listorti I Muzii L Amorini AM Longo S Di Stasio E. Low-molecular weight compounds in human seminal plasma as potential biomarkers of male infertility. Hum Reprod. (2018) 33:1817–28. doi: 10.1093/humrep/dey279 30239786

[31] Luedtke CC McKee MD Cyr DG Gregory M Kaartinen MT Mui J. Osteopontin expression and regulation in the testis, efferent ducts, and epididymis of rats during postnatal development through to adulthood. Biol Reprod. (2002) 66:1437–48. doi: 10.1095/biolreprod66.5.1437 11967208

[32] Cancel AM Chapman DA Killian GJ. Osteopontin is the 55-kilodalton fertility-associated protein in Holstein bull seminal plasma. Biol Reprod. (1997) 57:1293–301. doi: 10.1095/biolreprod57.6.1293 9408233

[33] Tekin K Kurtdede E Salmanoglu B Uysal O Stelletta C. Osteopontin concentration in prostates fractions: A novel marker of sperm quality in dogs. Vet Sci. (2023) 10:646. doi: 10.3390/vetsci10110646 37999469 PMC10675641

